# Supplementary material for: Complexome profiling of the Chlamydomonas psb28 mutant reveals TEF5 as an early PSII assembly factor
Source: Plant Cell. 2025 Mar 17;37(6):koaf055. doi: 10.1093/plcell/koaf055 (PMC12164590; doi:10.1093/plcell/koaf055)
Supplement: koaf055_Supplementary_Data [file koaf055_supplementary_data.zip › psb28_interactive.zip!/plots/intensity/qualityPlots/peptides/ACH1_Cre01.g042750.t1.2.html]

### Cre01.g042750.t1.2

Trivial name: ACH1  
SeqCoverage [median]: 52.50  
Mass: 89.15 kDa  
  
 

| Sequence | MissedCleavage | Mass [Da] | wtIntensity | muIntensity | Score |
| --- | --- | --- | --- | --- | --- |
| AGSALNAMAAAFK | 0 | 1221.62 | 140116.11 | 249984.28 | 293.53 |
| AIEDRLK | 1 | 843.48 | 0.00 | 2300.61 | 140.98 |
| AKVPFTISPGSEQIR | 1 | 1628.89 | 62718.15 | 382763.08 | 191.78 |
| APAASLADSFK | 0 | 1076.55 | 26850.60 | 71652.80 | 238.51 |
| APAASLADSFKEHLK | 1 | 1583.83 | 36429.44 | 208047.18 | 156.25 |
| AWNGDDIKDAAVLIK | 1 | 1627.86 | 4334.71 | 23284.33 | 111.74 |
| DAAVLIK | 0 | 728.44 | 0.00 | 7532.25 | 100.07 |
| DGIMDVFDK | 0 | 1038.47 | 50938.66 | 131263.88 | 196.99 |
| DTLVGADGK | 0 | 874.44 | 6841.39 | 25353.64 | 175.91 |
| EHAALEPR | 0 | 921.47 | 45929.06 | 85621.22 | 169.06 |
| EIKLEAPHGDELPSR | 1 | 1689.87 | 34246.32 | 100092.58 | 110.38 |
| EVYDFLATAGSK | 0 | 1299.63 | 13662.62 | 19289.11 | 198.62 |
| FAEEMR | 0 | 781.34 | 739.40 | 0.00 | 74.83 |
| FAEEMRK | 1 | 909.44 | 25052.01 | 41133.21 | 143.02 |
| FGGEADLSFAVK | 0 | 1239.61 | 25825.11 | 68912.18 | 239.39 |
| GEANSIITSFNR | 0 | 1307.65 | 9583.04 | 88978.64 | 272.43 |
| GFDAGANVYQAPPSSLDER | 0 | 1992.92 | 35278.30 | 201418.38 | 254.40 |
| GFDAGANVYQAPPSSLDERGK | 1 | 2178.03 | 2502.59 | 3671.01 | 107.65 |
| GHLDNISNNLLIGAINIENGKPNAVK | 1 | 2727.47 | 1691.09 | 11087.68 | 83.60 |
| GKLSVKVDPASQR | 2 | 1383.78 | 9871.44 | 8985.47 | 102.50 |
| GLPWVVVGDENYGEGSSR | 0 | 1919.90 | 6282.12 | 9355.04 | 135.04 |
| HLGGVAIIVK | 0 | 1005.63 | 68190.06 | 184090.03 | 158.12 |
| HLGGVAIIVKSFAR | 1 | 1466.87 | 0.00 | 7910.21 | 72.50 |
| IDPTDTISIVGLK | 0 | 1370.77 | 4092.40 | 5963.14 | 85.38 |
| IHETNLK | 0 | 853.47 | 9719.94 | 13302.39 | 130.77 |
| IHETNLKK | 1 | 981.56 | 7856.21 | 10026.19 | 143.61 |
| KRLNKPMTLAEK | 3 | 1427.83 | 3229.67 | 2064.46 | 71.08 |
| LEAPHGDELPSR | 0 | 1319.65 | 171305.88 | 250674.63 | 132.79 |
| LEAPHGDELPSRGFDAGANVYQAPPSSLDER | 1 | 3294.55 | 975.01 | 4672.62 | 63.49 |
| LNKPMTLAEK | 1 | 1143.63 | 105827.01 | 153758.68 | 163.90 |
| LQLLSPFK | 0 | 944.57 | 129862.99 | 246082.80 | 201.39 |
| LSVKVDPASQR | 1 | 1198.67 | 37574.35 | 49899.91 | 165.07 |
| MHDYLVATGR | 0 | 1161.56 | 79334.21 | 170638.81 | 135.63 |
| NGWPTELK | 0 | 943.48 | 2393.02 | 4902.11 | 85.21 |
| NVVTGAEGPVPATAR | 0 | 1437.76 | 262779.21 | 329440.27 | 322.57 |
| QALAAGIK | 0 | 770.47 | 117246.37 | 149478.82 | 148.98 |
| QGMLPVTFANPADYDK | 0 | 1765.83 | 0.00 | 1373.46 | 148.62 |
| QGMLPVTFANPADYDKIDPTDTISIVGLK | 1 | 3118.59 | 19548.25 | 0.00 | 146.17 |
| RLNKPMTLAEK | 2 | 1299.73 | 14407.79 | 23298.63 | 113.24 |
| RMHDYLVATGR | 1 | 1317.66 | 4497.84 | 11298.59 | 81.49 |
| RTDVPKGEANSIITSFNR | 2 | 2004.04 | 9103.42 | 25275.01 | 94.88 |
| SNKEVYDFLATAGSK | 1 | 1628.80 | 114562.30 | 350558.86 | 220.85 |
| TDVPKGEANSIITSFNR | 1 | 1847.94 | 100148.27 | 381975.85 | 204.90 |
| TFAPGVPLK | 0 | 928.54 | 53802.55 | 208346.00 | 119.29 |
| TFAPGVPLKVVGK | 1 | 1311.79 | 55150.85 | 104174.91 | 111.94 |
| VAGILTVK | 0 | 799.52 | 81167.78 | 159385.54 | 151.29 |
| VAMQDATAQMAMLQFISSGLPK | 0 | 2337.15 | 50128.28 | 141388.97 | 225.50 |
| VDPASQR | 0 | 771.39 | 76515.22 | 95419.64 | 166.86 |
| VPFTISPGSEQIR | 0 | 1429.76 | 94598.25 | 323474.11 | 343.19 |
| VPMSQFEPHEYLNDR | 0 | 1860.85 | 13422.46 | 44751.89 | 163.80 |
| VPMSQFEPHEYLNDRYK | 1 | 2152.00 | 7492.22 | 27864.38 | 156.49 |
| VVYGHLDDPENAEMK | 0 | 1715.78 | 5333.03 | 15245.16 | 100.69 |
| VVYGHLDDPENAEMKR | 1 | 1871.88 | 113933.54 | 174305.39 | 103.61 |
| WTSPKDVILK | 1 | 1185.68 | 3413.34 | 1520.63 | 81.77 |
| YRGHLDNISNNLLIGAINIENGKPNAVK | 2 | 3046.63 | 0.00 | 4970.63 | 61.42 |
|  |  |  | 2356503.9 | 5418259.3 |  |
